# A Multinational European Study of Patient Preferences for Novel Diagnostics to Manage Antimicrobial Resistance

**DOI:** 10.1007/s40258-019-00516-0

**Published:** 2019-09-20

**Authors:** David J. Mott, Grace Hampson, Martin J. Llewelyn, Jorge Mestre-Ferrandiz, Michael M. Hopkins

**Affiliations:** 1 Office of Health Economics, Southside, 7th Floor, 105 Victoria Street, London, UK; 2 Brighton and Sussex Medical School, Brighton, UK; 3 Independent Economics Consultant, Madrid, Spain; 4 Science Policy Research Unit, University of Sussex Business School, Jubilee Building, Falmer, Brighton, UK

## Abstract

**Background:**

Novel diagnostics are needed to manage antimicrobial resistance (AMR). Patient preferences are important in determining whether diagnostic tests are successful in practice, but there are few data describing the test attributes which matter most to patients. We elicited patients’ preferences for attributes of diagnostic tests that could be used to reduce unnecessary antibiotic use in primary care across seven European countries.

**Methods:**

We used an online stated preference survey, including a discrete choice experiment (DCE). The DCE explored how patients make trade-offs between three key attributes of diagnostic tests: the speed that results were available, confidence in the test results, and how convenient it is to take the test. Individuals were eligible to complete the survey if they had taken antibiotics within the last 2 years and were resident in Germany, Italy, Spain, France, Greece, the Netherlands or the United Kingdom (UK).

**Results:**

In total, 988 respondents completed the survey. The DCE responses illustrated that speed was the least important attribute in most countries. Responses from Germany and the Netherlands indicated that confidence was most important in these countries. Responses from the UK, France, Spain and Italy showed convenience as the most important attribute in these countries. Two attributes, confidence and convenience, were jointly favoured by respondents in Greece.

**Conclusion:**

Patients in different European countries do not have the same preferences for the attributes of diagnostic tests to manage AMR in primary care. Failure to account for such differences during test development could reduce test uptake, result in continued overuse of antibiotics, and hamper marketisation.

**Electronic supplementary material:**

The online version of this article (10.1007/s40258-019-00516-0) contains supplementary material, which is available to authorized users.

## Key Points for Decision Makers

**Table:** 

Patients in different European countries do not have the same preferences for the attributes of diagnostic tests aimed at managing antimicrobial resistance (AMR), indicating that different diagnostic tests might be more suitable for some European countries compared with others.
In the community setting, confidence in the test result was the most important attribute for patients in some countries, whereas the convenience of taking the test was the most important in others. The speed of obtaining a result was the least important attribute in all countries other than the UK.
Patient preferences should be considered when developing and providing diagnostic tests to manage AMR, as failure to offer acceptable tests in each market could lead to suboptimal uptake of testing and continued overuse of antibiotics, which is associated with higher levels of antibiotic resistance.

## Introduction

Growing antimicrobial resistance (AMR) in infectious diseases is a global health crisis. Antibiotic-resistant infections cause around 50,000 deaths each year across Europe and the USA, with many more in other areas of the world [1]. It has been estimated that by 2050, annual deaths from drug-resistant infections could reach 10 million globally unless effective action is taken [1]. Moreover, the World Bank have suggested that antibiotic-resistant infections have the potential to cause greater economic damage (in terms of an annual reduction in global GDP) than the 2008–2009 financial crisis [2]. AMR is highly problematic, because it limits the ability to treat common infections, ultimately leading to prolonged illnesses, increased costs, and death. Furthermore, availability of effective antibiotics makes many modern medical treatments possible such as surgical joint replacements, cancer chemotherapy and immunotherapies. At the same time, few new antibiotics are being developed to replace those that are becoming ineffective [3].

Development of novel diagnostic tests to minimise unnecessary antibiotic use has been identified as a key strategy to manage AMR. It is estimated that, with current diagnostic approaches, around 30% of antibiotic prescribing is unnecessary [4, 5]. The recent O’Neill review concluded that ‘rapid point-of-care diagnostic tests are a central part of the solution to this demand problem, which results currently in enormous unnecessary antibiotic use’ [6]. Initiatives such as the UK’s Longitude Prize and the Antimicrobial Resistance Diagnostic Challenge in the USA have been set up to drive innovation in diagnostics to help manage AMR.^1^

The development and uptake of diagnostics for AMR are challenging. Broadly speaking, there is a lack of investment by test developers, primarily due to expectations of low commercial returns—i.e. incentives for R&D are low and returns do not reflect the social value of controlling AMR through novel diagnostic approaches. On the demand side, inadequate education and behaviour of healthcare providers, among other factors, limits uptake of AMR diagnostics [6–8]. Beyond AMR, it has been reported that uptake of novel diagnostic tests more generally has been limited by local health service infrastructure, financial arrangements around reimbursement, or social factors related to clinician and patient resistance to use of new technology [9]. Indeed, Garau et al. highlight that pricing and reimbursement systems for diagnostics are inefficient because prices for diagnostics are often driven by administrative practices and expected production costs [10].

Patient perceptions are important when it comes to the use and misuse of antibiotics as the overuse of antibiotics is shaped significantly by physicians’ desire to satisfy patient demand [11]. It has been illustrated that parental expectations around antimicrobial prescribing drives clinicians’ prescription behaviour [12], and it has been suggested that joint physician-patient (or physician-carer) level decision making needs to be addressed in order to tackle the misuse of antibiotics [13]. These studies highlight the importance of patient engagement and support for reducing the misuse of  antibiotics. Despite the important role of patients in antibiotic misuse, there are currently no studies that examine patients’ preferences with respect to diagnostic tests that could mediate antibiotic use in this setting. Considering patient preferences when designing diagnostic tests is important because individuals’ preferences could directly influence their uptake of such tests, yet to date no studies have explored this issue.

We set out to bridge this important gap by eliciting patient preferences for public provision of diagnostic tests in this setting using a discrete choice experiment (DCE). DCE is a stated preference methodology that requires participants to make a series of hypothetical choices. The context for the research is the primary healthcare (community) setting, as this is where most antibiotic prescriptions are made; for instance, in the UK, it represented 81% in 2017 [14]. Patient views can shed light on the types of diagnostic tests that are most likely to be supported and will ultimately aid funders, developers and users in resource allocation and uptake planning to reduce the overuse and misuse of antibiotics. We set out to examine patient preferences in seven different counties: the five largest European economies [France (FR), Germany (DE), Italy (IT), Spain (ES) and the UK] plus the European countries with the highest and lowest use of antibiotics per inhabitant [Greece (GR) and the Netherlands (NL), respectively] [15].

## Methods

### Survey Overview

An online survey was designed, using the Qualtrics platform (http://www.qualtrics.com). The survey was developed in English and translated into the other six languages using a professional translation company (London Translations). To ensure translations were of high accuracy and maintained the intended meaning, these were checked and refined by members of the research team and/or academic colleagues with the requisite mother tongue.

The survey provided introductory information on AMR to give background and context to the study. Respondents were then introduced to the attributes for the DCE. Participants were asked if all of the important attributes were covered and, if not, to identify any missing attributes that they felt could be more important than those specified. Next, the participants undertook the DCE, followed by some feedback and background questions (these included demographics; previous use of antibiotics; prior awareness of AMR; willingness to pay for diagnostic testing; and comments on the survey questions). The survey instrument was designed for use in a range of settings and contained sections that are not explored in this article. The instrument is available as a supplemental material.

Ethical approval for the survey was sought and obtained from the University of Sussex (reference: ER/FL49/3).

### The Discrete Choice Experiment

#### Overview

DCE is a stated preference method, which presents respondents with a series of scenarios and requires them to make choices based on their preferences. In this study, the choice options are hypothetical diagnostic tests, which are described using a common set of characteristics referred to as ‘attributes’. Once respondents make a choice, they are presented with a new scenario containing two new tests, in which the attribute ‘levels’ of the new alternatives differ. The data from a sample of respondents can be used to determine the relative importance of the different attributes. In recent years, DCEs have become increasingly applied in health settings [16, 17].

#### Selection of Attributes and Levels

In order to identify the key attributes, a targeted literature review was carried out, focusing primarily on recent, policy-oriented initiatives and literature around desired characteristics for diagnostic tests used to manage the problem of AMR. Three key resources were identified as a starting point: the UK Government’s Review of diagnostics for AMR led by Lord O’Neill [1, 3, 4, 6], the ongoing UK-based Longitude Prize (http://www.longitudeprize.org) and a completed challenge “Horizon prize for better use of antibiotics” (European Commission).

The potential attributes and their definitions were discussed and refined with an advisory board made up of UK- and US-based representatives from diverse stakeholder groups. This board included a general practitioner; two medical microbiologists/hospital consultants in infectious diseases; two diagnostic industry representatives; and a representative of the lay public, all with special interest in infectious diseases and/or AMR. Care was taken to ensure that the labels given to the test attributes were suitable for the patient population, e.g. ‘confidence in test results’ rather than ‘specificity’ or ‘sensitivity’ of tests.

The advisory board also provided feedback on the levels of each attribute, to ensure that realistic levels were used. Choice was limited between a higher level of performance and a lower level of performance for each attribute. As the topic of diagnostics to manage AMR is already complex, it was deemed that making choices over a larger number of attributes and levels could become extremely cognitively demanding for respondents, and thus care was taken to keep the number of attributes and levels as low as possible, whilst still enabling the relevant trade-offs to be made.

The final attributes and levels for the DCE are shown in Table 1. Whilst many studies examine willingness to pay, cost was not included as an attribute in the DCE as it was not deemed appropriate in this context (most of the countries studied have largely publicly funded health care systems). Additionally, it has been shown that inclusion of a cost attribute can affect choice behaviour in DCEs, resulting in different relative preferences and an increase in error variance [18].

**Table 1:** Attributes and levels for the discrete choice experiment

Attribute	Definition	Levels
Speed at which results are available	Speed refers to the time between the sample being taken from the patient and the results becoming available to the healthcare professional	A fast test: results are available after 12 min A slow test: results are available the next working day
Convenience	Convenience refers to whether the use of a test requires clinical expertise and causes any discomfort for the patient	A test of high convenience: taking a sample does not require clinical expertise and does not cause discomfort for the patient A test of low convenience: taking a sample requires clinical expertise and causes discomfort for the patient
Confidence in the test result	Confidence is based on the test’s accuracy and reliability. Higher confidence in a test will make the result more influential on actual decision making by the user	A test in which the user has high confidence: there is an error rate of 10 in 100 A test in which the user has very high confidence: there is an error rate of 2 in 100

Each scenario contained two diagnostic tests and the setting for the scenario, which was in the community, was explicitly stated. Specifically, patients were asked which test should be made available to the health service. No opt-out option was provided (e.g. no test) as the intention was to examine relative importance of different characteristics of diagnostic tests, rather than to model uptake.

#### Experimental Design

**Fig. 1:**
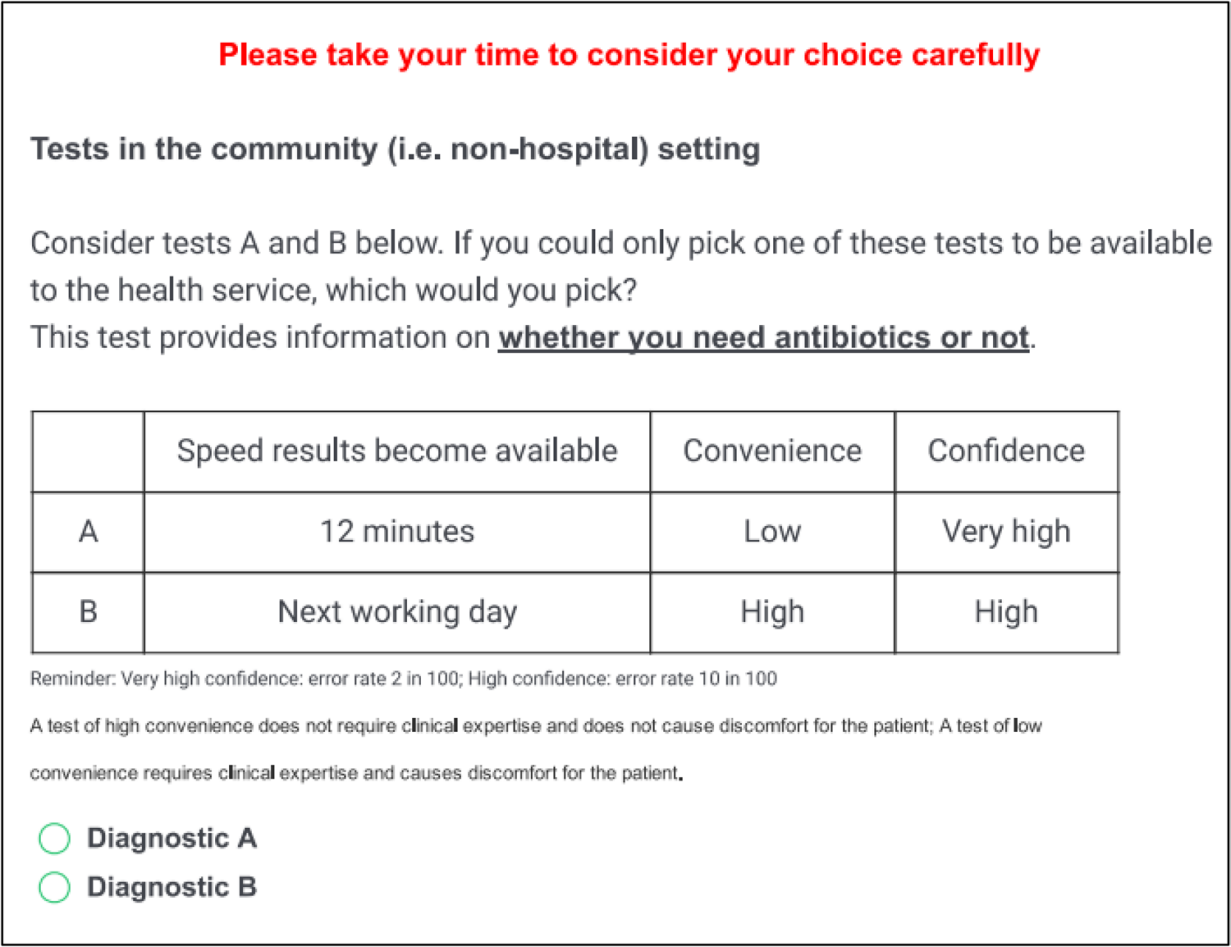
Example choice set

Given the relatively small number of attributes and levels (and no opt-out option), only four pairwise choice tasks were required to include every possible test within the design [19]. A scenario in which one test was intended to be ‘superior’ to the other across all attributes was included. The four pairwise tasks were presented in a random order to protect against ordering effects (such as learning, attention, or fatigue). We used an unlabelled design, with the tests in each choice being referred to simply as A and B. An example choice set can be found in Fig. 1, and the full survey can be found in the supplementary materials.

#### Piloting, Sampling and Data Collection

Once drafted, the survey was piloted amongst a small convenience sample of 30 lay contacts (e.g. colleagues, friends and family members of the project team). Feedback was subsequently incorporated into survey development. Respondents for the main survey were recruited and surveyed via an independent panel company. The panel company invited members of their existing panel via email and the respondents received a small financial incentive from the panel company for taking part. Patients were defined as people who had taken antibiotics in the past 24 months. Medical professionals were excluded from participating. All respondents were asked to confirm their consent via a click through page before beginning the survey. Based on further pilot testing with the panel provider and analysis of soft launch data (*n* = 125), we set a minimum response time of 3 min, and required patients to select their country of residence from a long list of countries. The country selected was required to be the same as the one being surveyed at the time to ensure participants were being diligent in their responses. Respondents who failed these measures were excluded from the dataset.

There is no universally accepted method for determining the necessary sample size for DCEs [20]. We identified several rules of thumb in the literature [21–23], and took the highest of these for our main analysis. We therefore obtained a minimum of 125 respondents from each country (minimum of 875 in total), based on the rule presented by Orme [22]. We considered this to be reasonable based on comparisons with other studies [20], and given the relatively small experimental design in our study.

#### Data Analysis

The selection of diagnostic test within each choice task was modelled as a function of the attributes and their levels using a random utility model framework. The utility obtained by respondent *n* choosing alternative *j* is given by Eq. 1.

1 $$V_{nj} = \beta_{1} {\text{ASC}}_{j} + \beta_{2} {\text{Speed}}_{j} + \beta_{3} {\text{Convenience}}_{j} + \beta_{4} {\text{Confidence}}_{j} ,$$

where ASC represents an alternative-specific constant (included to control for left-right bias) and the other independent variables represent the three attributes used within the DCE. The associated *β*s are the parameters estimated in the model.

The most commonly used choice model is the conditional logit (or multinomial logit), which was used as a starting point in this study [24]. However, the conditional logit has numerous well-documented limitations and more flexible models are often utilised [25]. In this study, mixed logit models were used in order to allow for random (unobservable) preference heterogeneity between respondents in each sample. All attributes from the DCE were modelled as random and normally distributed, and models were estimated using simulated maximum likelihood estimation via the *mixlogit* command in Stata 15.

As the *β* parameters are not directly interpretable, we focused on the ‘relative importance’ of each attribute and compared this information across countries. This was achieved by calculating the utility range for each attribute (based on the estimated parameters) and dividing these ranges by the total utility range (i.e. for all attributes). This provides percentages for each attribute that can be interpreted as the attribute’s importance. Potential issues when making comparisons between countries caused by differences in scale (see Vass et al. [26]) were avoided using this approach. Additional models with interactions based upon the respondent characteristics were also estimated to explore potential observable heterogeneity.

## Results

### Respondent Characteristics

A total of 988 individuals responded to the survey across the seven countries between April 2017 and April 2018. Sample sizes ranged from 126 in NL to 201 in GR. Respondents took just over 6.5 min (median) to complete the survey. However, a Kruskal–Wallis test indicated that there were statistically significant differences in median survey completion times between countries (*p* < 0.01; figures not reported). Respondents in GR had the largest median completion time (8.1 min) and respondents in the UK had the shortest median completion time (5.9 min).

Characteristics of the survey respondents are summarised in Table 2. In all samples, the most common age group was 21–40, although there was significant variation in the age bands between countries (*p* < 0.01). The proportion of males and females varied between samples (*p* < 0.01); however, in all samples female respondents predominated. Banded gross household income was recorded for all respondents in countries other than the UK (the UK survey was conducted first, and socioeconomic group was used; however, no comparable alternative was subsequently identified for the other countries). In these countries, respondents typically had a gross household income of less than €60,000, with significant variation across the income bands (*p* < 0.01). For example, in GR over half (55%) of the sample had a gross household income of under €20,000 whereas this was only the case for 12% of respondents in NL. In the UK, 66% of respondents fit into the higher socioeconomic group (ABC1), which indicates that the chief income earner in the respondent’s household works in a managerial, administrative or professional occupational group.

**Table 2:** Characteristics of the study population (*n* = 988)

	All countries		Germany		Spain		France		Greece		Italy		Netherlands		United Kingdom			*p* value^a,b,c^
	*n*	%	*n*	%	*n*	%	*n*	%	*n*	%	*n*	%	*n*	%	*n*		%	
*N*	988		139		131		129		201		127		126		135			
Age																		0.000
< 20 years	34	3	8	6	2	2	8	6	1	< 1	2	2	4	3	9		7	
21–40 years	483	49	63	45	82	63	66	51	114	57	58	46	50	40	50		37	
41–60 years	351	36	53	38	43	33	39	30	79	39	49	39	41	33	47		35	
61–80 years	119	12	15	11	4	3	16	12	7	3	18	14	31	25	28		21	
> 80 years	1	< 1	0	0	0	0	0	0	0	0	0	0	0	0	1		< 1	
Gender																		0.000
Male	430	44	53	38	58	44	51	40	94	47	58	46	57	45	59		44	
Female	558	56	86	62	73	56	78	60	107	53	69	54	69	55	76		56	
Income (all but UK)															SEG (UK only)			0.000
Up to €20,000	255	30	32	23	43	33	33	26	111	55	21	17	15	12	C2DE	35	26	
€20,000–€39,999	305	36	43	31	53	40	54	42	64	32	50	39	41	33	ABC1	89	66	
€40,000–€59,999	118	14	29	21	19	15	23	18	4	2	21	17	22	17	Unknown	11	8	
€60,000–€79,999	44	5	10	7	5	4	11	9	3	1	4	3	11	9				
€80,000 or more	33	4	9	6	3	2	3	2	1	< 1	9	7	8	6				
Don’t know/prefer not to say	98	11	16	12	8	6	5	4	18	9	22	17	29	23				
Previously aware of AMR	652	66	100	72	81	61	89	69	139	69	56	44	88	70		99	73	0.000
From media	375	38	63	45	40	31	35	27	82	41	33	26	54	43		68	50	
From friends and family	155	16	31	22	31	24	29	23	23	11	6	5	14	11		21	16	
From colleagues	87	9	17	12	8	6	17	13	15	7	7	6	9	7		14	10	
From medical professional	210	21	35	25	31	24	39	30	44	22	15	12	21	17		25	19	
From another source	56	6	5	4	9	7	5	4	10	5	8	6	13	10		6	4	
Not previously aware of AMR	336	34	39	28	50	38	40	31	62	31	71	56	38	30		36	27	
Frequency of antibiotic use																		0.000
1 infection in past two years	502	51	68	49	54	41	52	40	137	68	63	50	73	58		55	41	
> 1 infection in past two years	451	46	70	50	69	53	66	51	62	31	63	50	48	38		73	54	
Long-term, regular treatment	35	3	1	< 1	8	6	11	9	2	1	1	< 1	5	4		7	5	
Reason for recent antibiotic use																		0.000
Urinary infection	177	18	28	20	18	14	26	20	35	17	17	13	29	23		24	18	
Upper respiratory infection	322	33	38	27	58	44	52	40	85	42	50	39	20	16		19	14	
Intestinal	69	7	4	3	13	10	13	10	12	6	14	11	6	5		7	5	
Lower respiratory infection	203	20	39	28	17	13	19	15	30	15	28	22	23	18		47	35	
Sexually transmitted infection	19	2	2	1	0	0	2	2	6	3	1	1	6	5		2	1	
Other	198	20	28	20	25	19	17	13	33	16	17	13	42	33		36	27	

*AMR* antimicrobial resistance; *SEG* socio-economic group; *ABC1 and C2DE* can be considered as ‘middle class’ and ‘working class’, respectively

^a^*p* values related to *χ*^2^ tests

^b^Test for income/SEG involves income variable only (i.e. excludes UK)

^c^Test for AMR awareness compares ‘aware’ versus ‘not previously aware’ (i.e. does not include source of awareness)

In relation to AMR, respondents from the UK had the highest awareness overall (73%), respondents from IT had the lowest (44%) and there was significant variation across the various countries (*p* < 0.01). The most common source of awareness of AMR was the media in all countries other than FR, where medical professionals were the most common source. Very few respondents across the different countries used antibiotics for long-term, regular treatment (the highest was 9% in FR). For all samples other than GR and NL, 50% or more of the respondents indicated that they had taken antibiotics more than once in the past 2 years. The difference in proportions across these three categories of antibiotic use frequency was statistically significant (*p* < 0.01). The most common reason for the most recent use of antibiotics was upper respiratory infections in ES, FR, GR and IT; lower respiratory infections in UK and DE, and other (various) infections in NL. The differences between the samples were statistically significant (*p* < 0.01).

### Analysis of Choice Data

Prior to completion of the DCE, respondents were asked whether they considered speed, convenience and confidence to be the three most important attributes in a diagnostic test for AMR. Overall, 96% of respondents said yes (minimum 92% in DE; maximum 98% in ES). There was little consensus amongst those responding negatively on possible additional attributes.

**Fig. 2:**
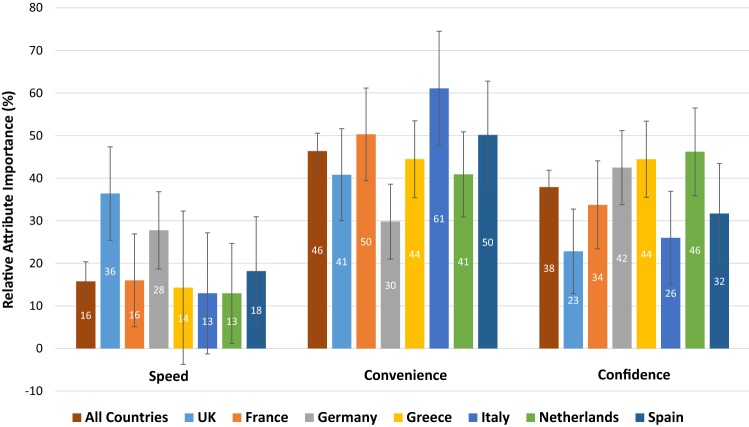
Relative importance of the attributes. Notes: All estimates of relative importance were statistically significant at the 1% level, with the exception of: speed in Greece (not significant at the 10% level); speed in Italy (significant at the 10% level); and speed in The Netherlands (significant at the 5% level)

The results of the mixed logit models are shown in Table 3, and the relative importance of each attribute (based on the output from the mixed logit models) is illustrated in Fig. 2. Speed was the least important attribute in every sample except the UK, and the speed coefficients in the GR, IT and NL samples were statistically insignificant (indicating that, on average, this attribute was not influential when respondents made their choices). The most important attribute varied between convenience and confidence depending on the country. Confidence was the most important attribute in both the NL and DE samples, with convenience the most important in the UK, FR, IT and ES samples. In the GR sample, both convenience and confidence appear to be equally important. The standard deviation estimates in Table 3 indicate that preferences for each attribute often differed within samples to a significant extent.

**Table 3:** Regression output from mixed logit models

	All countries	UK	France	Germany	Greece	Italy	Netherlands	Spain
Constant	0.155** (0.044)	0.103 (0.134)	0.178 (0.110)	0.112 (0.115)	0.095 (0.097)	0.208 (0.120)	0.132 (0.113)	0.443* (0.187)
Speed	0.319** (0.058)	0.950** (0.245)	0.281* (0.117)	0.648** (0.159)	− 0.226 (0.131)	0.240 (0.155)	0.245 (0.132)	0.534* (0.247)
Convenience	0.940** (0.073)	1.065** (0.253)	0.883** (0.168)	0.696** (0.160)	0.906** (0.159)	1.131** (0.218)	0.773** (0.155)	1.474** (0.401)
Confidence	0.769** (0.063)	0.595** (0.180)	0.592** (0.138)	0.992** (0.178)	0.906** (0.152)	0.481** (0.138)	0.873** (0.168)	0.932** (0.298)
SD (speed)	1.096** (0.113)	1.598** (0.406)	0.383 (0.371)	0.927** (0.282)	1.176** (0.260)	1.067** (0.301)	0.712* (0.287)	1.814** (0.564)
SD (convenience)	1.015** (0.112)	1.466** (0.381)	0.734** (0.277)	0.842** (0.284)	0.951** (0.256)	1.012** (0.304)	0.506 (0.331)	1.982** (0.575)
SD (confidence)	0.775** (0.114)	0.964** (0.359)	0.533 (0.304)	0.689* (0.299)	0.689* (0.269)	0.376 (0.438)	0.642* (0.298)	1.689** (0.536)
Observations	7904	1080	1032	1112	1608	1016	1008	1048
Log-likelihood	− 2386.5	− 319.5	− 308.1	− 323.5	− 482.7	− 300	− 298.2	− 309.9

Mixed logit models using 1000 Halton draws, with all variables except the constant modelled as random and normally distributed; standard errors in parentheses

*SD* standard deviation

**p < 0.01, *p < 0.05

Coefficients for all attributes in Table 3 were positive (with the exception of speed in the GR sample, although this was not statistically significant), indicating that respondents prefer faster, more convenient tests in which the user can have greater confidence. Overall 78% of respondents selected the ‘superior’ test (minimum 74% GR; maximum 84% DE) in the choice set that contained one test that was superior to the other across all attributes.

Additional choice models were also estimated containing a range of interactions in order to determine whether observable demographic characteristics influence respondents’ preferences. The characteristics were: gender (male/female); age (up to 40/over 40 years); and AMR awareness (aware/unaware). Models containing three attribute interactions were estimated for each country, for each characteristic (21 models in total). If the model contained a statistically significant interaction term (at the 5% significance level) and had a better model fit relative to a model without interactions (using likelihood ratio tests with 5% significance), the interaction was considered to be important. This was the case for four models. In summary, females in the GR sample appear to have less of a preference for speed relative to males whereas in the IT sample, relative to males, females had a lesser preference for convenience. In the DE sample, respondents aged > 40 years had a lesser preference for speed relative to those aged ≤ 40. Finally, in the IT sample, individuals who were previously aware of AMR had a higher preference for speed relative to those who were not previously aware.

A single mixed logit model was also estimated containing all observations for all countries; these results are reflected in Table 3 and Fig. 2. Overall, the combined model illustrates that, when the data are pooled, convenience was the most important attribute followed by confidence, with speed the least important.

## Discussion

### Our Findings and Previous Studies

As far as we are aware, this is the first multi-national analysis of patient preferences for diagnostic tests for AMR. As such, directly comparable literature is not available. Nonetheless, differences in patient preferences between countries have also been found in various health settings, such as primary care [27], anti-osteoporosis treatments [28] and medication persistence [29]. In addition, there have been numerous studies that examine patient preferences for other diagnostic tests, such as screening tests for: cancer [30]; Alzheimer’s disease [31]; colorectal cancer [32]; Down’s syndrome [33] and leukaemia [34]. These studies provide additional evidence to suggest that patients value convenience [31], as well as additional evidence of the importance of confidence in diagnostic test results [30, 32–34]. Notably, two of these studies (focused on tests for Down’s syndrome and leukaemia) suggest that confidence is more important than waiting time [33, 34], which is in line with our findings. However, it is important to note the potential differences in the clinical importance of test speed between this study on AMR and others in the literature. In many cases where antibiotics may be required, it is feasible that there could be a significant benefit in the short-term, and a quick result might be desirable for rapid prescription decisions. In addition, in the primary care setting there is perhaps less risk to life if a diagnostic test for infection provides a false result as compared to the studies above. Therefore, the finding that speed was not particularly important to respondents in many of the study countries is somewhat surprising. Whilst this may be a robust finding, it could be explained if respondents were not really convinced that waiting for a test result would in reality delay their access to antibiotics. Given the current easy access to these drugs in the countries studied and the lack of routine diagnostic testing, the scenario presented would have been unfamiliar.

It is interesting to note that, compared with the findings of the Eurobarometer, a significantly smaller percentage of respondents, in all of our samples, expressed that they were previously aware of AMR [35]. The rates of awareness in the former ranged from being 14–26 percentage points higher than the rates that we observed. It may be the case that the use of the full term ‘antimicrobial resistance’ was new to respondents, whereas the idea that antibiotics become less effective as use increases was broadly understood (this is more aligned with the wording used in the Eurobarometer).

### Implications for Managing AMR

Taking the pragmatic assumption that no single diagnostic test is likely to be optimal in all attributes, it becomes important to understand the priorities of patients. Patients play a central role when it comes to the uptake of diagnostic tests, and developers, clinicians and policymakers therefore need to be aware of these priorities. Our results suggest that in the community setting, accurate test results and convenient testing are typically higher priorities for patients than the speed with which results become available. Additionally, the identification of differences in preferences between countries is also important because it suggests that a ‘one size fits all’ approach, even within Western Europe, might not be effective.

### Strengths and Limitations

To our knowledge this is the first study eliciting patient preferences for diagnostic tests for AMR. It provides pan-European data based on trade-offs, which advances our understanding of the types of diagnostic tests that are most likely to be acceptable to patients. This information could be valuable in the design and marketing of such diagnostics and policies to encourage their use. This could ultimately help to reduce the overuse and misuse of antibiotics.

However, this study does have its limitations. For example, the sample populations were generally younger and more female-dominated relative to the general populations of each country [36]. This could be a reflection of individuals who have taken antibiotics in the past 2 years (inclusion criteria for the study), or a reflection of the online panel characteristics. It should also be noted that whilst we elicited preferences from experienced patients (i.e. those who had received antibiotics recently), funding decisions are often made based on general population preferences [37, 38]. The advantage is that our sample will be more informed than the general population, but the disadvantage is that the results may not be representative of the general populations of the study countries. Ultimately, our choice of sample reflects a pragmatic decision over a normative one.

Other limitations relate to the study design. Whilst 96% of respondents agreed that speed, convenience and confidence were the most important attributes, it may be the case that respondents preferred to passively agree rather than to actively disagree, putting faith in our decision to focus on these attributes. Another limitation is that only 78% of respondents chose the ‘superior’ diagnostic test in the scenario where one alternative was dominant over the other. However, whilst many studies observe ‘pass rates’ of 90% or higher [39], some observe similar pass rates to ours such as the 79% pass rate observed by Gerard et al. [40]. We were unable to conduct a typical robustness test—running a model with only those that passed and determining if the results differ—due to collinearity issues caused by the small experimental design and thus cannot determine the potential impact of this result. Finally, another limitation is the use of normal distributions for the random parameters in the mixed logit models. In theory, it may have been more appropriate to use log-normal distributions, as we had no a priori expectations that any respondents would prefer slower, less convenient tests or less accurate tests. However, models with random parameters modelled with log-normal distributions failed to converge. Nonetheless, as we wanted to account for unobservable heterogeneity, we preferred to use mixed logit models with normally distributed random parameters over conditional logit models.

### Further Research

Having explored patients’ preferences for different attributes of diagnostics to manage AMR, another important step could be to explore the preferences of other stakeholder groups, including clinicians, nurses and pharmacists, whose preferences may also be influential in the uptake of diagnostics to manage AMR. Further research beyond the European countries studied here would also be potentially useful to stakeholders concerned with the management of AMR in different contexts. Particularly in the developing world, where the threat of AMR is particularly great [41], there may be a need for greater understanding of patient preferences. Specifically, knowledge of whether stakeholder group or nationality is the stronger influence on preferred test attributes, and in particular whether doctors and patients share the same preferences, could be crucially useful in attempting to encourage uptake of diagnostic tests to manage AMR. Additionally, preferences may differ depending on the setting (e.g. primary or secondary care) and depending on the exact context (i.e. why antibiotics were required and seriousness of disease, for example). Finally, respondents have been asked to consider preferences in regard their own treatment. However, preferences may vary in the case of parents requesting treatment for young children and infants.

## Conclusion

This study of diagnostic tests to manage AMR in the primary care setting suggests that typically convenience and confidence, rather than speed, are the important attributes for patients when undergoing tests in the community to detect whether an antibiotic prescription is warranted. However, the results of this study illustrate that patients’ preferences differ between these European countries. Therefore, stakeholders cannot assume that the preferences of patients for diagnostic tests to manage AMR will be similar across European markets.

These results may help to inform the development and provision of diagnostic tests that will be acceptable to patients across Europe. This is important because failure to produce acceptable tests in each market could lead to suboptimal uptake and use, with continued overuse of antibiotics as a result. Such overuse is associated with higher levels of antibiotic resistance.

## Electronic supplementary material

Below is the link to the electronic supplementary material.

Supplementary material 1 (DOCX 34 kb)

## Data Availability

The datasets generated during and/or analysed during the current study are available from the corresponding author on reasonable request.

## Footnotes

1 See https://www.longitudeprize.org/ and https://dpcpsi.nih.gov/AMRChallenge for more information.
