# Supplementary material for: A Multinational European Study of Patient Preferences for Novel Diagnostics to Manage Antimicrobial Resistance
Source: Appl Health Econ Health Policy. 2019 Sep 20;18(1):69–79. doi: 10.1007/s40258-019-00516-0 (PMC6978300; doi:10.1007/s40258-019-00516-0)
Supplement: Supplementary file 1 — Supplementary material 1 (DOCX 34 kb) [file 40258_2019_516_MOESM1_ESM.docx]

**Applied Health Economics and Health Policy**

**A multinational European study of patient preferences for novel diagnostics to tackle antimicrobial resistance**

Mott, D.J.^*^, Hampson, G., Llewelyn, M.J., Mestre-Ferrandiz, J., Hopkins, M.M.

^*^ Corresponding author. Affiliation: Office of Health Economics, London, UK; Email: [dmott@ohe.org](mailto:dmott@ohe.org)

**Supplementary Material – Online Survey**

The following contains the survey questions used to generate the dataset for the article “*A multinational European study of patient preferences for novel diagnostics to tackle antimicrobial resistance*”.

Originally there were two variants of the survey, and respondents self-selected one option:

1. Tests in a hospital setting
2. Tests in a community setting

The former (hospital setting) was removed post-launch due to poor uptake and a slight change of focus. Therefore, any questions pertaining to this variant of the survey have been deleted using tracked changes in this document for clarity.

MRC AMR - Lay - 12/04/2017

Start of Block: Introduction

**Choosing Medical Tests to Manage Resistance to Antibiotics: An International Survey**

Antibiotics are important medicines useful for preventing and treating bacterial infections. However, in recent years bacteria have begun to survive treatment with antibiotics on a worrying scale. This ‘antimicrobial resistance’ to antibiotics is now a significant threat to patients' safety across the world.

To control antimicrobial resistance it is essential to avoid misuse of antibiotics. One way to ensure that antibiotics are more carefully used is to use diagnostic tests (or simply "tests") to help doctors decide which patients need antibiotics.

This survey will ask you to make choices about the type of tests that you think would be most helpful in reducing the misuse of antibiotics.

By asking a wide range of people to make these choices, the survey will reveal the attributes that tests will need to have to be an acceptable tool for the control of antimicrobial resistance.

| Page Break |  |
| --- | --- |

**Please answer all questions with yourself in mind, rather than anyone you care for.**
The survey is intended to take no more than 10 minutes and it is easiest to complete it in one go. 

This survey is being conducted by the University of Sussex and the Office for Health Economics - both of which are not-for-profit research organisations.

The survey is sponsored by the ‘Tackling Antimicrobial resistance’ collaborative research initiative, funded by the UK Government’s research councils.

We are not conducting research on behalf of a commercial organisation.

If you have any specific accessibility requirements, such as requiring a hard copy of this survey, please email ghampson@ohe.org.

| Page Break |  |
| --- | --- |

| 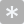 |
| --- |

Participation in this survey is voluntary. All responses will be anonymised.

The findings of the survey will be reported publically and used to inform policy debate. Further information on the survey, data use and protection is available here: [Additional information](https://universityofsussex.eu.qualtrics.com/CP/File.php?F=F_8csKqiR04O0I00B%22%20target=%22_blank)

Before you participate, please read each of the following statements and then **confirm that you agree** by **clicking the confirmation statement** below:

I confirm that I have read and understood the information above.

I understand that my annonymous response will be used for research and to inform policy debate.

I consent to the use of my annonymous response for the above purposes.

- Click here to confirm you have been adequately informed about the survey and agree with the three statements above. (1)

| Page Break |  |
| --- | --- |

How often do you take antibiotics?

- I receive long term treatment with antibiotics to prevent infections (1)
- I have received treatment with antibiotics for more than one infection in the last two years (2)
- I have received treatment with antibiotics for one infection in the last two years (3)
- I have not taken antibiotics in the past two years (4)

| Page Break |  |
| --- | --- |

End of Block: Introduction

Start of Block: Block 12

Start of Block: Ranking exercise COMMUNITY

We will ask you to make choices about diagnostic tests which have particular attributes. These attributes are explained below. 

**Speed** at which results are available

Speed refers to the time between the sample being taken from the patient and the results becoming available to the healthcare professional.

**Convenience**

Convenience refers to whether the use of a test requires clinical expertise and causes any discomfort for the patient

**Confidence** in the test result

Confidence refers to the user's understanding of the test's accuracy and reliability. The higher the user's confidence in a test, the more it will influence their decision making.

Do you consider confidence, convenience and speed to be the top 3 most important attributes for a test that is used to determine whether you need antibiotics or not **in the community (i.e. non-hospital) setting?**

- Yes (1)
- No (2)

Display This Question:

If Do you consider confidence, convenience and speed to be the top 3 most important attributes for a... = No

Please identify any attributes which you consider to be more important than those listed here (specifically for a test that is used to determine whether you need antibiotics or not):

________________________________________________________________

| Page Break |  |
| --- | --- |

End of Block: Ranking exercise COMMUNITY

Start of Block: DCE description

You will now be presented with a series of questions in which you are asked to choose between two hypothetical diagnostic tests, each with different attributes. In each question you will be asked to choose between diagnostic A and diagnostic B, but the tests presented will differ between questions.

You will be asked to provide your views on tests that provide information on whether or not you need an antibiotic.

| **Attribute** | **Definition and choices** |
| --- | --- |
| **Speed** at which results are available | Speed refers to the time between the sample being taken from the patient and the results becoming available to the healthcare professional.  **You will be asked to choose between:**  **A fast test:** Results are available after 12 minutes  **A slow test:** Results are available the next working day. |
| **Convenience** | Convenience refers to whether the use of a test requires clinical expertise and causes any discomfort for the patient.  **You will be asked to choose between:**  **A test of high convenience:** Taking a sample does not require clinical expertise and does not cause discomfort for the patient  **A test of low convenience:** Taking a sample requires clinical expertise and causes discomfort for the patient. |
| **Confidence** in the test result | Confidence is based on the test's accuracy and reliability. Higher confidence in a test will make the result more influential on actual decision making by the user.  **You will be asked to choose between:**  **A test in which the user has high confidence:** There is an error rate of 10 in 100  **A test in which the user has very high confidence:** There is an error rate of 2 in 100. |

Please note that we will ask you at the end of the survey whether you have answered these questions with a specific illness and/or setting in mind.

End of Block: DCE description

Start of Block: COMMUNITY CHOICES illness

**Please take your time to consider your choice carefully**
**Tests in the community (i.e. non-hospital) setting**

Consider tests A and B below. If you could only pick one of these tests to be available to the health service, which would you pick?  This test provides information on **whether you need antibiotics or not**. 

|  | **Speed results become available** | **Convenience** | **Confidence** |
| --- | --- | --- | --- |
| **Diagnostic A** | 12 minutes | High | High |
| **Diagnostic B** | Next working day | Low | Very high |

Reminder:

Very high confidence: error rate 2 in 100; High confidence: error rate 10 in 100;
A test of high convenience does not require clinical expertise and does not cause discomfort for the patient; A test of low convenience requires clinical expertise and causes discomfort for the patient.

- Diagnostic A (1)
- Diagnostic B (2)

| Page Break |  |
| --- | --- |

**Please take your time to consider your choice carefully**
 **Tests in the community (i.e. non-hospital) setting**   Consider tests A and B below. If you could only pick one of these tests to be available to the health service, which would you pick?  This test provides information on **whether you need antibiotics or not**. 

|  | **Speed results become available** | **Convenience** | **Confidence** |
| --- | --- | --- | --- |
| **Diagnostic A** | 12 minutes | Low | Very High |
| **Diagnostic B** | Next working day | High | High |

Reminder:

Very high confidence: error rate 2 in 100; High confidence: error rate 10 in 100;
A test of high convenience does not require clinical expertise and does not cause discomfort for the patient; A test of low convenience requires clinical expertise and causes discomfort for the patient.

- Diagnostic A (1)
- Diagnostic B (2)

| Page Break |  |
| --- | --- |

**Please take your time to consider your choice carefully**
 **Tests in the community (i.e. non-hospital) setting**   Consider tests A and B below. If you could only pick one of these tests to be available to the health service, which would you pick?  This test provides information on **whether you need antibiotics or not**. 

|  | **Speed results become available** | **Convenience** | **Confidence** |
| --- | --- | --- | --- |
| **Diagnostic A** | Next working day | High | Very high |
| **Diagnostic B** | 12 minutes | Low | High |

Reminder:

Very high confidence: error rate 2 in 100; High confidence: error rate 10 in 100;
A test of high convenience does not require clinical expertise and does not cause discomfort for the patient; A test of low convenience requires clinical expertise and causes discomfort for the patient.

- Diagnostic A (1)
- Diagnostic B (2)

| Page Break |  |
| --- | --- |

**Please take your time to consider your choice carefully**
 **Tests in the community (i.e. non-hospital) setting**   Consider tests A and B below. If you could only pick one of these tests to be available to the health service, which would you pick?  This test provides information on **whether you need antibiotics or not**. 

|  | **Speed results become available** | **Convenience** | **Confidence** |
| --- | --- | --- | --- |
| **Diagnostic A** | Next working day | Low | High |
| **Diagnostic B** | 12 minutes | High | Very high |

Reminder:

Very high confidence: error rate 2 in 100; High confidence: error rate 10 in 100;
A test of high convenience does not require clinical expertise and does not cause discomfort for the patient; A test of low convenience requires clinical expertise and causes discomfort for the patient.

- Diagnostic A (1)
- Diagnostic B (2)

| Page Break |  |
| --- | --- |

End of Block: COMMUNITY CHOICES illness

Start of Block: Feedback

Do you have any further comments on the choices you have been asked to make?

________________________________________________________________

________________________________________________________________

________________________________________________________________

________________________________________________________________

________________________________________________________________

When you were choosing between tests A and B in the previous questions did you have a specific healthcare situation in mind?

- Yes (1)
- No (2)

Display This Question:

If When you were choosing between tests A and B in the previous questions did you have a specific he... = Yes

What sort of infection were you thinking about?

- Urinary infection (e.g. kidney / bladder) (1)
- Lower respiratory infection (e.g. chest infection) (2)
- Upper respiratory infection (e.g. throat or ear) (3)
- Intestinal (e.g. stomach bug) (4)
- Sexually transmitted infection (5)
- Other (please specify) (8) ________________________________________________

Display This Question:

If When you were choosing between tests A and B in the previous questions did you have a specific he... = Yes

And In the following sections you will be asked to answer questions on your preferences regarding tes... = Community (i.e. non-hospital)

What setting of community care were you thinking about?

- Patient's own home (1)
- Care or residential home (2)
- Community pharmacy (3)
- General Practitioner or family doctor surgery (4)
- Other (please specify) (5) ________________________________________________

End of Block: Feedback

Start of Block: WTP (community)

Consider a test that is highly convenient, users have very high confidence in the results (error rate 2 in 100) and results are delivered within 12 minutes. 
This test would be used in the community (i.e. non-hospital) setting to determine whether it is appropriate for you to be prescribed antibiotics, and could therefore be an important step in tackling the problem of resistance to antibiotics.
Suppose that the health service is unable to cover the cost of the test. In which case, you would be required to pay a fee each time you needed to use the test, on top of any other charges that you currently pay for healthcare.
For each of the values below, please indicate whether you would or would not be willing to pay the amount stated for the test to be provided:

|  | Yes (1) | No (2) |
| --- | --- | --- |
| £1 (2) |  |  |
| £5 (3) |  |  |
| £10 (4) |  |  |
| £50 (5) |  |  |
| £100 (6) |  |  |
| £1,000 (7) |  |  |
| More than £1,000 (please specify) (8) |  |  |

Display This Question:

If Consider a test that is highly convenient, users have very high confidence in the results (error... = £1 [ No ]

Why are you not willing to pay for this test?

- I cannot afford to pay (1)
- The test is not suitable (2)
- The government should pay (3)
- Other (please specify) (4) ________________________________________________

End of Block: WTP (community)

Start of Block: background questions LAY

Please indicate your awareness of the issue of antimicrobial resistance and the problems that this raises for healthcare. You can select one or more options

- I was not aware of this issue before this survey (1)
- I was aware of this issue being discussed in the media (2)
- I was aware of this issue from communication with friends and family (3)
- I was aware of this issue from communication with colleagues at work (4)
- I was aware of this issue from communication with a medical professional (5)
- I have previously participated in a research study that has made me aware of this issue (6)
- I was aware of this issue through other communication (please specify) (7) ________________________________________________

| Page Break |  |
| --- | --- |

Are you:

- Male? (1)
- Female? (2)

Which age category do you fall in to?

- <20 years (1)
- 21-40 years (2)
- 41-60 years (3)
- 61-80 years (4)
- >80 years (5)

Which country do you currently live in?

- Austria (2)
- France (3)
- Germany (4)
- Italy (5)
- Spain (6)
- Poland (7)
- Greece (8)
- Cyprus (14)
- Netherlands (9)
- Ireland (10)
- Belgium (11)
- Romania (27)
- Luxembourg (25)
- Portugal (13)
- Denmark (15)
- Finland (16)
- Sweden (17)
- Bulgaria (20)
- United Kingdom (12)
- Croatia (21)
- Czech Republic (18)
- Estonia (19)
- Hungary (22)
- Latvia (23)
- Lithuania (24)
- Malta (26)
- Slovakia (28)
- Slovenia (29)

| Page Break |  |
| --- | --- |

How would you describe the chief income earner's occupation type before retirement?

- Senior managerial or professional (1)
- Intermediate managerial, administrative or professional (2)
- Supervisor, clerical, junior managerial, administrative or professional (3)
- Manual worker (with industry qualifications) (4)
- Manual worker (with no qualifications) (5)
- None of these (6)

Display This Question:

If How would you describe the occupation type of the chief income earner in your household? = Retired

Does the chief income earner have a state pension, a private pension or both?

- State only (1)
- Private only (2)
- Both (3)

Display This Question:

If Does the chief income earner have a state pension, a private pension or both? = Private only

Or Does the chief income earner have a state pension, a private pension or both? = Both

How would you describe the occupation type of the chief income earner in your household?

- Senior managerial or professional (1)
- Intermediate managerial, administrative or professional (2)
- Supervisor; clerical; junior managerial, administrative or professional (3)
- Manual worker (with industry qualifications) (4)
- Manual worker (with no qualifications) (5)
- Unemployed more than 6 months (if less than 6 months select based on previous status) (6)
- Retired (7)
- Student (8)
- Prefer not to say (9)

| Page Break |  |
| --- | --- |

Did you last receive antibiotics:

- During a stay in hospital? (1)
- Following a visit to hospital? (2)
- Without referral to a hospital? (3)

In which country did you most recently receive antibiotics?

- France (1)
- Germany (2)
- Greece (3)
- Italy (4)
- Netherlands (5)
- Spain (6)
- UK (7)
- Other (please specify) (8) ________________________________________________

Most recently, why did you need to take antibiotics?

- Urinary infection (e.g. kidney / bladder) (1)
- Lower respiratory infection (e.g. chest infection) (2)
- Upper respiratory infection (e.g. throat or ear) (3)
- Intestinal (e.g. stomach bug) (4)
- Sexually transmitted infection (5)
- Other (please specify) (8) ________________________________________________

Do you have any further comments on the questions you have answered?

________________________________________________________________

________________________________________________________________

________________________________________________________________

________________________________________________________________

________________________________________________________________

End of Block: background questions LAY
